# MS can be considered a primary progressive disease in all cases, but some patients have superimposed relapses – Yes

**DOI:** 10.1177/13524585211001789

**Published:** 2021-04-20

**Authors:** Antonio Scalfari

**Affiliations:** Centre of Neuroscience, Department of Medicine, Imperial College London, Charing Cross Hospital, London, UK

Only few multiple sclerosis (MS) patients escape the progressive course if they survive long enough. In the majority of cases, the progression supervenes after a variable latency from the onset of the relapsing remitting phase, but the clinical boundaries between the relapsing and the progressive course are often indistinct. There are no surrogate markers or universally accepted definition for the continuous unremitting disability accumulation and defining the clinical phenotypes of MS can be challenging even for experienced clinicians.

The current phenotypical distinction of relapsing remitting (RR), secondary progressive (SP) and primary progressive (PP) MS provides a standardized terminology, which enhances homogeneity in clinical trials. However, despite the variable patterns of evolution, there are no biological reasons for discerning different phenotypes. Pathological mechanisms underlying acute attacks and progression are known to be tightly intermingled and to occur concomitantly since the early phase of the disease, irrespective of clinical symptoms. The same wide spectrum of central nervous system tissue alterations is seen across all stages, with only quantitative, rather than qualitative differences.^1^ Both PP and SP MS share similar pathological features in respect of the extent of inflammatory infiltrates, axonal damage and cortical demyelination.^1^ Epidemiological data corroborate a unifying disease model of MS, with predominantly age-related clinical phenotypes. By growing older, the risk of experiencing a progressive course proportionally increases, while relapses gradually become sparse and infrequent.^2^ Progressive MS patients, with or without a preceding RR course, accumulate disability at very similar rate and share strikingly similar mean age at onset of progression, indicating that PPMS is preceded by an ‘asymptomatic RR phase’.^3^ Consistent with this view, subjects with radiologically isolated syndrome can develop a PP course years after the incidental detection of white matter abnormalities, confirming a protracted pre-progressive prodrome with subclinical disease activity similar to RRMS.^4^

Despite being biologically active, progressive MS can remain clinically undetectable for years. Most clinicians would be reluctant to document a progressive accumulation of disability unrelated to clinical attacks during the early stage of the RR phase. Based on the recently proposed objective definition of SPMS, the attainment of at least expanded disability status scale (EDSS) of 4 is required to mark the transition to the progressive phase.^5^ However, the clinical progression can be uncovered in the early phase of the disease, among patients with no permanent motor impairment, especially when relapses with poor recovery, which can potentially cloud the progressive accumulation of disability, are uncommon. This was highlighted by George Ebers’ group by addressing the clinical course of single attack progressive MS, which is a rare phenotype distinguished by a single demyelinating episode at onset, followed some years later by the progressive phase. Because of the lack of ongoing relapses, in this subgroup, the progression mostly presented clinically with exercise-induced ambulation worsening and could be pinpointed after 8 mean years from onset, at an average EDSS of 2, therefore much earlier than in classic SPMS cases.^6^

In line with these early intuitive observations, recent data indicate that a continuous progression independent of relapsing activity (PIRA) is commonly observed during the RR phase. The pooled analysis of the two OPERA trials demonstrated, in both the Interferon and Ocrelizumab groups with a relatively short disease duration (mean 6 years) and low baseline mean EDSS score (= 2.8), an impressively large proportion of patients (78% and 87%, respectively) accumulating disability, which was unrelated to inflammatory attacks and was mostly secondary to worsening of the walking impairment.^7^ This provides clinical evidence of an early underlying progressive course despite the effective therapeutic relapse suppression and should caution against using the lack of ongoing focal inflammation as marker of disease stability. The adequate control of inflammatory parameters might provide a sense of false security, while a continuous smouldering process underpins the subtle clinical deterioration, which stands out as an important unmet treatment target. Changes in the whole brain and grey matter volume,^7^ the accumulation of chronic slowing expanding lesions^8^ and the microglia activation, both in the normal appearing white matter and in the perilesional areas,^9^ are plausible drivers of PIRA events during the RR phase, when the focal inflammatory activity is the dominant clinical feature, albeit representing only the tip of the pathological iceberg.

These observations challenge the dichotomy between relapsing and progressive disease, supporting a one stage disorder model of MS, where all patients exhibit a progressive course since the disease onset, which can be overlapped by relapses. Detrimental processes, spanning across all disease stages and underpinning the progressive accumulation of disability, start early and subtly emerge clinically under the strong influence of age-related biological changes, which induce immune senescence and exhaustion of compensatory mechanisms.^10^ The individual immune system proactivity accounts for the simultaneous highly variable superimposed relapsing activity, which overlaps the progressive course and additionally contributes to the disability accumulation. In this wide spectrum of age-related clinical manifestations, young patients are more likely to experience relapse onset progressive MS and display an initial floridly inflammatory course, which gradually subsides by growing older, while the subclinical neurodegeneration becomes clinically evident. At the opposite extreme lay older patients, who present with a PP course and are distinguished by a resistance to the clinical inflammatory activity, possibly related to undetermined immunological qualitative differences, compared to SPMS.

Alike other neurodegenerative disorders, such as Parkinson or Alzheimer diseases, progressive MS is preceded by a prodromal phase of unknown duration before meeting its conventional clinical definition. It remains debated whether relapses represent a concomitant epiphenomenon to the primary neuroaxonal loss, which potentially promotes the release of highly antigenic myelin fragments, secondarily triggering the innate and adaptive immune responses. This inside-out model would contrast with the traditional outside-in view of a primary process, starting in the periphery with a dysregulated immune reaction and causing an inflammatory response, which eventually lead to the axonal degeneration. The tight interconnection between neurodegenerative and inflammatory mechanisms makes the two hypotheses equally likely, leaving the question unresolved. However, evidence of early progressive accumulation of disability independent of relapses discloses a dissociation between the therapeutic suppression of the inflammatory activity and the disease progression. This highlights the need of shifting our treatments strategies to target more broadly all pathological mechanisms driving MS, with a specific focus on halting smouldering processes, which account for the progressive worsening since the early stage.
